# Cost Effectiveness of Ranibizumab vs Aflibercept vs Bevacizumab for the Treatment of Macular Oedema Due to Central Retinal Vein Occlusion: The LEAVO Study

**DOI:** 10.1007/s40273-021-01026-5

**Published:** 2021-04-26

**Authors:** Becky Pennington, Abualbishr Alshreef, Laura Flight, Andrew Metry, Edith Poku, Philip Hykin, Sobha Sivaprasad, A. Toby Prevost, Joana C. Vasconcelos, Caroline Murphy, Joanna Kelly, Yit Yang, Andrew Lotery, Michael Williams, John Brazier

**Affiliations:** 1 School of Health and Related Research, University of Sheffield, Sheffield, UK; 2 NIHR Moorfields Biomedical Research Centre, London, UK; 3 Nightingale-Saunders Clinical Trials and Epidemiology Unit at King’s Clinical Trials Unit, King’s College London, London, UK; 4 King’s Clinical Trials Unit at King’s Health Partners, King’s College London, London, UK; 5 Wolverhampton Eye Infirmary, Wolverhampton, UK; 6 Faculty of Medicine, University of Southampton, Southampton, UK; 7 Centre for Medical Education, Queen’s University of Belfast, Belfast, UK

## Abstract

**Background:**

We aimed to assess the cost effectiveness of intravitreal ranibizumab (Lucentis), aflibercept (Eylea) and bevacizumab (Avastin) for the treatment of macular oedema due to central retinal vein occlusion.

**Methods:**

We calculated costs and quality-adjusted life-years from the UK National Health Service and Personal Social Services perspective. We performed a within-trial analysis using the efficacy, safety, resource use and health utility data from a randomised controlled trial (LEAVO) over 100 weeks. We built a discrete event simulation to model long-term outcomes. We estimated utilities using the Visual-Functioning Questionnaire-Utility Index, EQ-5D and EQ-5D with an additional vision question. We used standard UK costs sources for 2018/19 and a cost of £28 per bevacizumab injection. We discounted costs and quality-adjusted life-years at 3.5% annually.

**Results:**

Bevacizumab was the least costly intervention followed by ranibizumab and aflibercept in both the within-trial analysis (bevacizumab: £6292, ranibizumab: £13,014, aflibercept: £14,328) and long-term model (bevacizumab: £18,353, ranibizumab: £30,226, aflibercept: £35,026). Although LEAVO did not demonstrate bevacizumab to be non-inferior for the visual acuity primary outcome, the three interventions generated similar quality-adjusted life-years in both analyses. Bevacizumab was always the most cost-effective intervention at a threshold of £30,000 per quality-adjusted life-year, even using the list price of £243 per injection.

**Conclusions:**

Wider adoption of bevacizumab for the treatment of macular oedema due to central retinal vein occlusion could result in substantial savings to healthcare systems and deliver similar health-related quality of life. However, patients, funders and ophthalmologists should be fully aware that LEAVO could not demonstrate that bevacizumab is non-inferior to the licensed agents.

**Supplementary Information:**

The online version contains supplementary material available at 10.1007/s40273-021-01026-5.

## Key Points for Decision Makers

**Table:** 

Although bevacizumab was not non-inferior to ranibizumab and aflibercept in LEAVO, the three interventions generate similar quality-adjusted life-years.
Bevacizumab is always the most cost-effective intervention at £20,000–£30,000 per quality-adjusted life-year.

## Introduction

Macular oedema (MO) due to central retinal vein occlusion (CRVO) is associated with vision-related quality-of-life impairment and costs to healthcare systems and societies more broadly [1, 2]. The annual incidence of visual impairment from MO due to CRVO in England and Wales is estimated to be 5700 [3–6]. Central retinal vein occlusion may be ischaemic or non-ischaemic, with ischaemic CRVO being associated with further complications such as neovascular glaucoma [7]. The prevalence and incidence of CRVO increase with age [3].

Aflibercept (Eylea) [2 mg/0.05 mL (Bayer Pharma AG)], ranibizumab (Lucentis) [0.5 mg/0.05 mL (Novartis)] and bevacizumab (Avastin) [1.25 mg/0.05 mL (Roche)] are anti-vascular endothelial growth factor inhibitors given by a repeated intravitreal injection to treat MO due to CRVO. Aflibercept and ranibizumab are licensed for this indication [8, 9] with list prices per injection of £816 and £551, respectively [10, 11]. The National Institute for Health and Care Excellence (NICE) recommends ranibizumab and aflibercept (each with a discount on the list price) as treatments for MO due to CRVO [12, 13].

Bevacizumab, currently available off-label for this indication, costs £243 per large vial, or £28 per injection when separated from the vial into pre-filled syringes [14, 15]. Because of the potential for cost savings, bevacizumab has been proposed as an alternative intervention for MO due to CRVO [1]. The Court of Appeal recently ruled that offering off-label bevacizumab to National Health Service (NHS) patients with wet age-related macular degeneration is lawful [16]. Our aim was to compare the cost effectiveness of bevacizumab, ranibizumab and aflibercept for treating MO due to CRVO.

## Methods

### LEAVO Study

LEAVO was a multicentre, randomised non-inferiority clinical trial of 463 (non-ischaemic: 406, ischaemic: 56, missing ischaemic status: 1) participants conducted in 44 UK NHS hospitals, comparing ranibizumab, aflibercept and bevacizumab for the treatment of MO due to CRVO [17, 18]. The primary outcome was change in best-corrected visual acuity (BCVA) Early Treatment Diabetic Retinopathy Study letter score (number of letters read on a chart at a fixed distance) from baseline to 100 weeks. The clinical effectiveness analysis was unable to demonstrate that bevacizumab was non-inferior (non-inferiority limit defined as − 5 Early Treatment Diabetic Retinopathy Study letters) to ranibizumab in the intention-to-treat (ITT) population (adjusted mean BCVA difference − 1.73 letters; 95% confidence interval [CI] − 6.12 to 2.67; *p* = 0.071). Aflibercept was non-inferior to ranibizumab in the ITT population (adjusted mean BCVA difference 2.23 letters; 95% CI − 2.17 to 6.63; *p* = 0.0006) but not superior. A post hoc analysis was unable to show that bevacizumab was non-inferior to aflibercept in the ITT population (adjusted mean BCVA difference − 3.96 letters; 95% CI − 8.34 to 0.42; *p* = 0.32). The per-protocol results were similar [18].

### Economic Evaluation Overview

We conducted a within-trial analysis using individual patient-level data from LEAVO to calculate the costs and quality-adjusted life-years (QALYs) over 100 weeks and a decision analytic model to calculate the costs and QALYs over the entire lifetime horizon [19]. We considered the NHS and Personal Social Services perspective, in accordance with the NICE Methods Guide [20]. We discounted costs and QALYs at 3.5% annually [20]. We compared results using an incremental analysis, as preferred by NICE [20]. We calculated the probability that bevacizumab was the most cost-effective intervention at £20,000 and £30,000 per QALY. The methods for the health economic analysis were pre-specified prior to the database lock [19].

### Data Analysis

#### Within-Trial

We used an ITT analysis, including all the participants randomised to each treatment group. When a participant withdrew from the study, and a withdrawal appointment occurred, we assigned their cost and utility data to the nearest visit, all subsequent costs were set to zero and recorded utilities as missing. If there was no withdrawal appointment, subsequent costs and utilities were assumed to be missing at random. We used multiple imputation using chained equations with predictive mean matching to impute missing values of costs, QALYs and baseline covariates to account for missing data [21].

We used a seemingly unrelated regression model to estimate the difference in mean total costs and QALYs between treatment arms, taking into account correlation [22, 23]. The regression equation for total costs included the randomisation arm. The regression equation for QALY included the randomisation arm and baseline utility to control for imbalances between treatment arms [22, 23]. In the model, we assumed a normal distribution for both costs and QALYs. We calculated marginal effects in each treatment arm using the seemingly unrelated regression without adjusting for baseline utility.

#### Economic Model

We constructed a discrete event simulation to model the pathway of individual patients through a set of events from the beginning of LEAVO until death, according to the time sampled for each event [24]. The advantages of a discrete event simulation in this application were:

1. Health states were not required, thus each individual patient’s visual acuity could be tracked over time on a continuous scale.
2. The study eye and non-study eye could be modelled separately.
3. Each patient’s history could be tracked, to allow incorporation of the treatment continuation rule (see Sect. 2.4.2).
4. The follow-up visit times could be modelled using the treatment continuation rule and LEAVO milestone visit schedule (see Sect. 2.4.2).
5. Individual patients could have different baseline characteristics to incorporate heterogeneity.

**Fig. 1:**
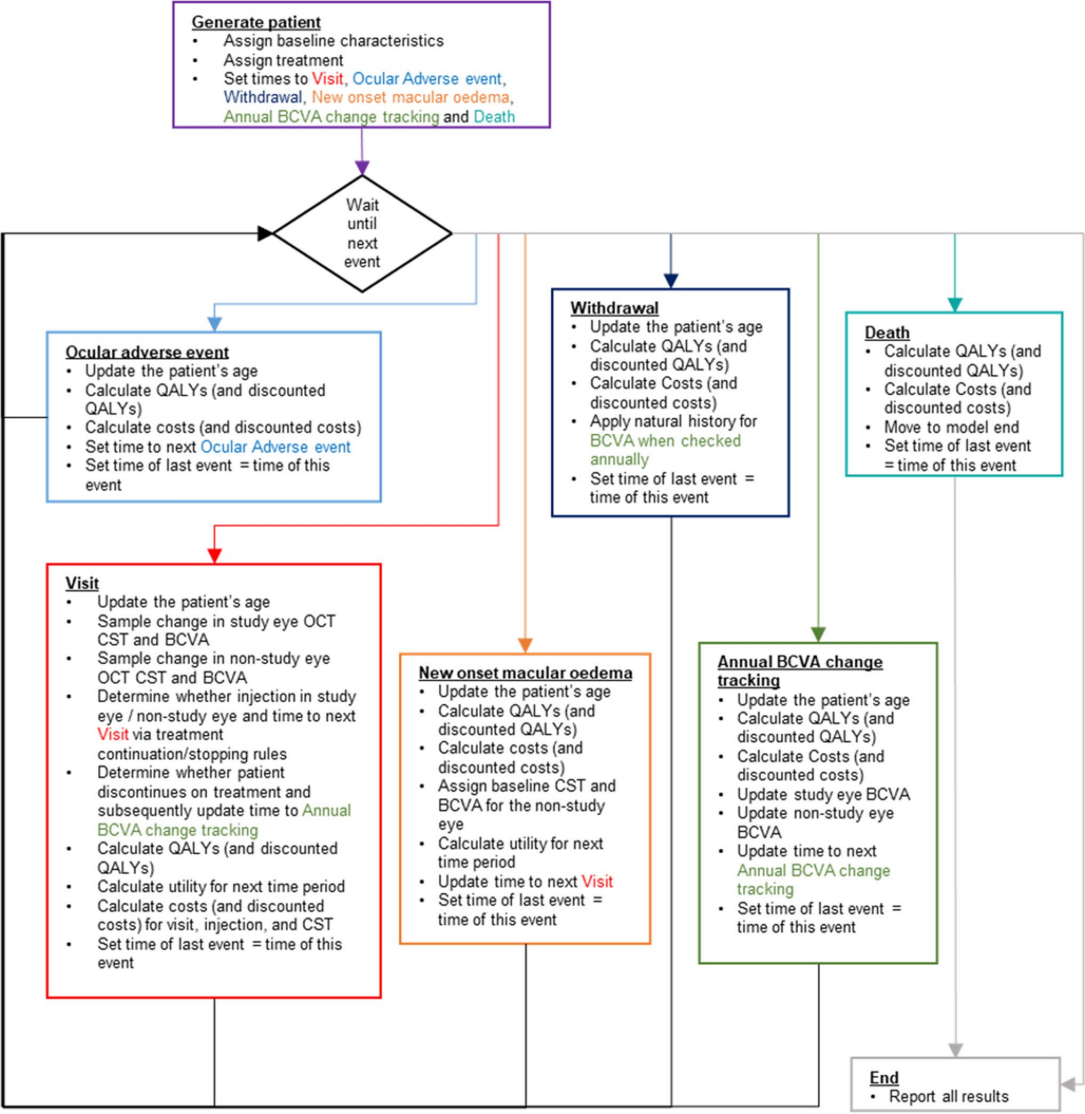
Model diagram. *BCVA* best-corrected visual acuity, *CST* central subfield thickness, *OCT* optical coherence 5 tomography, *QALY* quality-adjusted life-year

Events in the model were visits to an ophthalmologist (to assess and administer injections), ocular adverse events, withdrawal, new-onset MO in the non-study eye and death. We assigned sampled times to different events each time a patient was simulated and updated them as necessary (see Fig. 1), allowing the patient’s history to influence when and whether future events occurred. Simulated patients moved to the next chronological event, where their visual acuity, costs and utility were updated. We assumed that the baseline characteristics of LEAVO patients (age, sex and visual acuity) were representative of the MO due to CRVO population in England, and randomly assigned an entire patient profile for simulated patients to preserve the relationship between baseline characteristics. We included development of MO in the non-study eye at an annual rate of 0.009 per year (calculated by fitting an exponential distribution to the eight LEAVO patients who developed MO in their non-study eye) and assumed that patients would receive the same intervention in both eyes. We simulated 7000 patients for each intervention, as the total costs and QALYs had stabilised by this point. A full list of input parameters and stabilisation graphs are provided in the Electronic Supplementary Material (ESM).

### Data Sources

#### Health Utilities

LEAVO included three measures of health-related quality of life (HRQoL) at each visit: the Visual Functioning Questionnaire-Utility Index (VFQ-UI) [25], the five-level EQ-5D (a generic measure used across disease areas and preferred by NICE) [20] and the EQ-5D with an additional vision dimension (EQ-5D V) [26]. These measures are used to estimate “utility”, where 1 is full health and 0 is equivalent to death, and can then be combined with life-years to calculate QALYs. In the within-trial analysis, we used the utilities at each visit for each patient to calculate total QALYs using linear interpolation. As the model predicted BCVA beyond the trial period (see Sect. 2.4.8), to convert BCVA to utility in the economic model, we developed statistical models that predicted HRQoL as a function of BCVA in both eyes, age and sex [27]. As specified in the Health Economics Analysis Plan prior to conducting the study [19], we used VFQ-UI in the base case to model utility as EQ-5D has been shown to perform poorly in eye conditions [28], and EQ-5D and EQ-5D V in scenario analyses.

#### Intervention Costs

In LEAVO, the treatment regime included mandated injections of ranibizumab, aflibercept or bevacizumab at weeks 0, 4, 8 and 12 followed by visits at weeks 16 and 20 when they could receive an injection if their study eye BCVA was ≤ 83 letters, and they met at least one of the retreatment criteria:

- Decrease in visual acuity of ≥ 6 letters between the previous and current visit and an increase in central subfield thickness, or
- Increase in visual acuity of ≥ 6 letters between the previous and current visit, or
- Central subfield thickness (CST) > 320 µm, or
- CST increase of > 50 µm from the lowest previous visit.

The retreatment criteria were applied between weeks 24 and 96. If a patient did not meet retreatment criteria at three consecutive visits, the visit interval was increased from 4 to 8 weeks. In the within-trial analysis, costs associated with delivering the intervention included drug costs, appointment costs and test costs including: optical coherence tomography, colour fundus photography and fundus fluorescein angiography.

The model included the mandated injections for all patients and simulated the same retreatment criteria and included treatment withdrawal modelled separately for the three interventions (see ESM). We sought advice from five clinicians (PH, SS, YY, AL, MW) and guidance from the Royal College of Ophthalmologists [3] for modelling ophthalmologist visits beyond the trial period and assumed that after 100 weeks:

- Patients who had not had injections after 52 weeks in LEAVO no longer received injections or visited the ophthalmologist to be assessed.
- Between 100 weeks and 5 years, we applied the same retreatment criteria as in LEAVO, but increased the time between visits to 12 weeks, and assumed that patients who were not treated at three consecutive visits no longer received injections or visited the ophthalmologist to be assessed.
- Beyond 5 years, we assumed that patients no longer received injections but had three follow-up visits with the ophthalmologist, 12 weeks apart. We included the costs of the injections and the costs of visits to the ophthalmologist.

#### Resource Use

We included resource use costs associated with MO due to CRVO, collected in LEAVO using a specially developed resource use questionnaire at baseline, 12, 24, 52, 76 and 100 weeks (given in the ESM) in the trial-based analysis. We estimated linear regression models using ordinary least squares to predict the number of appointments with different healthcare professionals as a function of BCVA in the worse-seeing eye, and used these in the model to extrapolate healthcare resource use beyond the trial period.

#### Unit Costs

The list prices for ranibizumab (£551 per injection) and aflibercept (£816 per injection) were from the British National Formulary [10, 11]. In LEAVO, bevacizumab vials were compounded into pre-filled syringes at the Royal Liverpool and Broadgreen University Hospitals Pharmacy Aseptic Unit, costing £28 per injection [15], which the Medicines and Healthcare products Regulatory Agency clarified does not create an unlicensed medicine [29]. We assumed the cost per injection includes any costs associated with compounding the drug, such as staff time and storage costs. We used the list price of £243 for bevacizumab in a scenario analysis, equivalent to assuming a vial can only be used for a single injection with the remainder of the vial wasted [14]. We used 2018 NHS Reference Costs and Unit Costs of Health and Social Care for resource use where possible, uplifting older costs to 2018 using hospital and community health services indices [30, 31]. Unit costs are shown in Table 1.

**Table 1:** Unit costs

Parameter	Used in within-trial analysis or model	Mean (standard error)	Distribution used in model	Reference (mean)	Reference (standard error)
Ranibizumab injection	Both	£551.00	N/A	BNF 2019 [5]	N/A
Aflibercept injection	Both	£816.00	N/A	BNF 2019 [6]	N/A
Bevacizumab injection	Both	£28.00	N/A	Judicial review (2018) [10]	N/A
Central subfield thickness examination	Both	£108.21 (£1.70)	Gamma	NHS Improvement (2018) [26] NHS codes BZ87A	Quartile data of the NHS codes Department of Health (2017) [41]
First visit to ophthalmologist	Both	£140.04 (£9.91)	Gamma	NHS Improvement (2018) [26] NHS codes WF02B	
Follow-up visit to ophthalmologist	Both	£105.19 (£4.88)	Gamma	NHS Improvement (2018) [26] NHS codes WF02A	
Optical coherence tomography	Within-trial	£116.23	N/A	NHS Improvement (2018) [26] NHS codes BZ89A	N/A
Colour fundus photograph	Within-trial	£108.21	N/A	NHS Improvement (2018) [26] NHS codes BZ87A	N/A
Fundus fluorescein angiograph	Within-trial	£108.21	N/A	NHS Improvement (2018) [26] NHS codes BZ87A	N/A
Accident and emergency visit	Both	£160.23 (£9.34)	Gamma	NHS Improvement (2018) [26] Weighted average for NHS codes VB01Z to VB11Z	Quartile data of the NHS codes (weighted) Department of Health (2017) [41]
Ocular accident and emergency visit	Both	£118.02 (£2.67)	Gamma	NHS Improvement (2018) [26] NHS codes WF01B	
Eye consultant visit	Both	£95.13 (£1.85)	Gamma	NHS Improvement (2018) [26] NHS codes WF01A	
Ophthalmologist call	Both	£28.20 (£4)	Gamma	NHS Improvement (2018) [26] NHS codes WF01D	
Optometrist/optician visit	Both	£76.50 (£10.5)	Gamma	NHS Improvement (2018) [26] NHS codes WF01B	
Low vision appointment visit	Both	£153.00	N/A	Estimated to be double the visit cost of an optometrist/optician to reflect additional complexity (on clinician advice)	
General practitioner visit	Both	£37.40 (£3.74)	Gamma	Curtis and Burns (2018) [25]	10% assumption around the mean
Practice nurse visit	Both	£17.79 (£1.78)	Gamma		
General practitioner call	Both	£28.00 (£2.8)	Gamma		
Community care (annual)	Model	£10,060.95 (£1006.10)	Gamma	Curtis and Burns (2018) [25]	10% assumption around mean
Hip replacement (annual)	Model	£4170.00 (£417.00)	Gamma	NHS Improvement (2018) [24] Code HT14C	10% assumption around mean
Low vision aids (one-off)	Both	£194.41 (£19.44)	Gamma	Meads (2003), Curtis and Burns (2018)	10% assumption around mean
Low vision rehabilitation (one-off)	Model	£153	Gamma	Estimated to be double the visit cost of an optometrist/optician	
Residential care (annual)	Model	£6000.80 (£600.08)	Gamma	Curtis and Burns (2018) [25]	10% assumption around mean
Treatment for depression (annual)	Model	£2430.58 (£243.06)	Gamma	NICE, 2017 (TA460) [42]	10% assumption around mean
Blindness registration (one-off)	Both	£60.50 (£6.05)	Gamma	Curtis and Burns (2018) [25]	10% assumption around mean
Adverse event	Model	£317.96 (£28.62)	Gamma	NHS Improvement (2018) [26]	Weighted variance from NHS reference costs

*BNF* British National Formulary, *N/A* not applicable, *NHS* National Health Service, *NICE* National Institute for Health and Care Excellence

#### Blindness Costs

We included a cost of blindness, which is made up of one-off costs for blind registration for all patients, and low vision aids and low vision rehabilitation for a proportion of patients (33% and 11%) in the model and trial-based analysis. The model additionally included annual costs for proportions of patients requiring community care (6%), residential care (30%), treatment for depression (39%) and hip replacement (5%) [32]. Blindness costs were sourced using NHS Reference Costs and Unit Costs of Health and Social Care for resource use [30, 31] and the resource use from published economic evaluations in ophthalmic indications [32] as the duration of LEAVO was not long enough to collect reliable estimates for blindness costs. The model included blindness costs for patients whose BCVA in both eyes was below 35 letters (rare in CRVO) [33], consistent with previous models in MO [12, 13]. The within-trial analysis included costs for partial visual impairment for patients whose BCVA in both eyes was less than or equal to 58 letters (≤ 6/24) in both eyes and severe visual impairment if their BCVA was less than or equal to 19 letters (< 3/60) in both eyes. The within-trial analysis aimed to reflect the highest possible costs associated with blindness, assuming the same proportion of partially sighted patients would register as blind and incur the same costs as those who are severely sight impaired.

#### Ocular Adverse Events

We included costs for ocular adverse events in the within-trial analysis using data from the resource use questionnaire, case report forms and concomitant procedure and medication logs. In the model, we included ophthalmic adverse events based on the frequency in LEAVO using an average NHS cost (see ESM) [24]. Simulated patients could have more than one ophthalmic adverse event.

#### Mortality

In the within-trial analysis, when a patient died, we set their utility scores at all subsequent visits to zero, and assumed that half the costs expected between the previous and next scheduled visit were incurred. This was to reflect the fact the participants may have incurred costs between visits before they died. In the model, we incorporated mortality by applying age- and sex-specific standardised mortality ratios to UK lifetable data to reflect the additional mortality associated with CRVO [34, 35]. Modelled patients who died no longer incurred costs or QALYs. None of the LEAVO participants died as a consequence of the treatments and thus there was no loss of life costs incurred.

#### Visual Acuity

We fitted equations to LEAVO data to predict BCVA for the first 100 weeks of starting treatment as a function of baseline BCVA, age, intervention, number of injections, the time since the most recent injection and time-variant covariates at weeks 12, 24, 52 and 76 weeks (see Appendix in the ESM). Beyond 76 weeks, log-likelihood tests indicated models without the time-varying covariates should not be rejected and can therefore be used to extrapolate beyond 100 weeks. Similar equations to predict CST were fitted as required for the retreatment criteria (see ESM). For untreated eyes (including non-study eyes, and those that withdrew from or discontinued treatment), we modelled changes in BCVA annually using data from the Beaver Dam study [36].

### Base-Case and Scenario Analyses

#### Within-Trial

For the VFQ-UI base case, we used a parametric approach to address the uncertainty around the cost-utility analyses estimates. We calculated the probability of each treatment being the most cost effective by sampling the mean costs and QALYs from a bivariate normal distribution. We additionally conducted a complete case analysis, excluding patients with any missing data and a 52-week analysis using imputed data up to the 52-week milestone visit.

#### Economic Model

We estimated the percentage discount required for aflibercept and ranibizumab to be cost effective compared to bevacizumab at the £20,000 per QALY threshold. We additionally considered a 100-week time horizon for validation against the within-trial analysis.

For the base-case and scenario analyses, we undertook a probabilistic sensitivity analysis, simultaneously sampling all uncertain parameters from their distributions (see Appendix in the ESM). We presented means and 95% CIs for total and incremental costs and QALYs and the mean ICER. We ran 500 simulations, a number sufficient to avoid decision uncertainty.

## Results

### Within-Trial Analysis

#### Base Case

A total of 462 participants were included in the within-trial analysis (one participant was excluded as no data were provided). Bevacizumab was the least costly intervention (£6292), followed by ranibizumab (£13,014) and aflibercept (£14,328). Bevacizumab was statistically significantly cheaper than ranibizumab and aflibercept (Table 2).

**Table 2:** Within-trial analysis: base-case and scenario analysis results

	Total (SD)		Incremental (95% CI)		ICER (£)
	Costs (£)	QALYs	Costs (£)	QALYs^a^	
*Base-case analysis*					
Bevacizumab	6292 (5759–6824)	1.666 (1.629–1.704)	–	–	
Ranibizumab	13,014 (12,444–13,584)	1.627 (1.588–1.666)	6734 (5970–7498)	− 0.019 (− 0.066 to 0.028)	Dominated
Aflibercept	14,328 (13,731–14,925)	1.651 (1.613–1.690)	7984 (7209–8759)	− 0.015 (− 0.062 to 0.032)	Dominated
*Scenario analysis: EQ-5D for utilities*					
Bevacizumab	6273 (5738–6808)	1.535 (1.476–1.595)	–	–	
Ranibizumab	13,068 (12,493–13,643)	1.513 (1.454–1.572)	6769 (5987–7550)	− 0.010 (− 0.071 to 0.050)	Dominated
Aflibercept	14,271 (13,661–14,882)	1.560 (1.499–1.619)	8035 (7246–8824)	0.008 (− 0.053 to 0.068)	1,041,476
*Scenario analysis: EQ-5D V for utilities*					
Bevacizumab	6268 (5736–6800)	1.500 (1.441–1.5591)	–	–	
Ranibizumab	13,000 (12,421–13,579)	1.472 (1.414–1.530)	6748 (5948–7547)	− 0.035 (− 0.117 to 0.048)	Dominated
Aflibercept	14,273 (13,684–14,861)	1.516 (1.455–1.577)	8012 (7232–8793)	0.003 (− 0.084 to 0.090)	2,483,943
*Complete case analysis*					
Bevacizumab	6459 (5587–7332)	1.651 (1.603–1.699)			
Ranibizumab	12,608 (11,756–13,461)	1.656 (1.609–1.703)	6149 (4929–7369)	0.007 (− 0.046 to 0.060)	890,736
Aflibercept	14,013 (13,167–14,859)	1.691 (1.644–1.737)	1405 (204–2606)	0.011 (− 0.041 to 0.063)	130,020
*Scenario analysis: 52* *weeks*					
Bevacizumab	3621 (3302–3940)	0.884 (0.866–0.903)	–	–	
Ranibizumab	8164 (7822–8506)	0.865 (0.845–0.884)	4565 (4085–5045)	− 0.008 (− 0.030 to 0.014)	Dominated
Aflibercept	9214 (8860–9568)	0.880 (0.861–0.899)	5560 (5082–6039)	− 0.004 (− 0.026 to 0.017)	Dominated
*Scenario analysis: bevacizumab list price from BNF (£243)*					
Bevacizumab	8933 (8384–9482)	1.666 (1.629–1.704)	–	–	
Ranibizumab	13,014 (12,433–13,595)	1.627 (1.588–1.666)	4093 (3281–4904)	−0.019 (− 0.066 to 0.028)	Dominated
Aflibercept	14,328 (13,721–14,935)	1.651 (1.613–1.690)	5342 (4552–6133)	− 0.015 (− 0.062 to 0.032)	Dominated

*BNF* British National Formulary, *CI* confidence interval, *EQ*-*5D* EuroQol-5 Dimension, *EQ*-*5D V* EuroQol-5 Dimension with Vision bolt-on, *ICER* incremental cost-effectiveness ratio, *QALY* quality-adjusted life-year, *SD* standard deviation

^a^Adjusted for baseline utility score

**Fig. 2:**
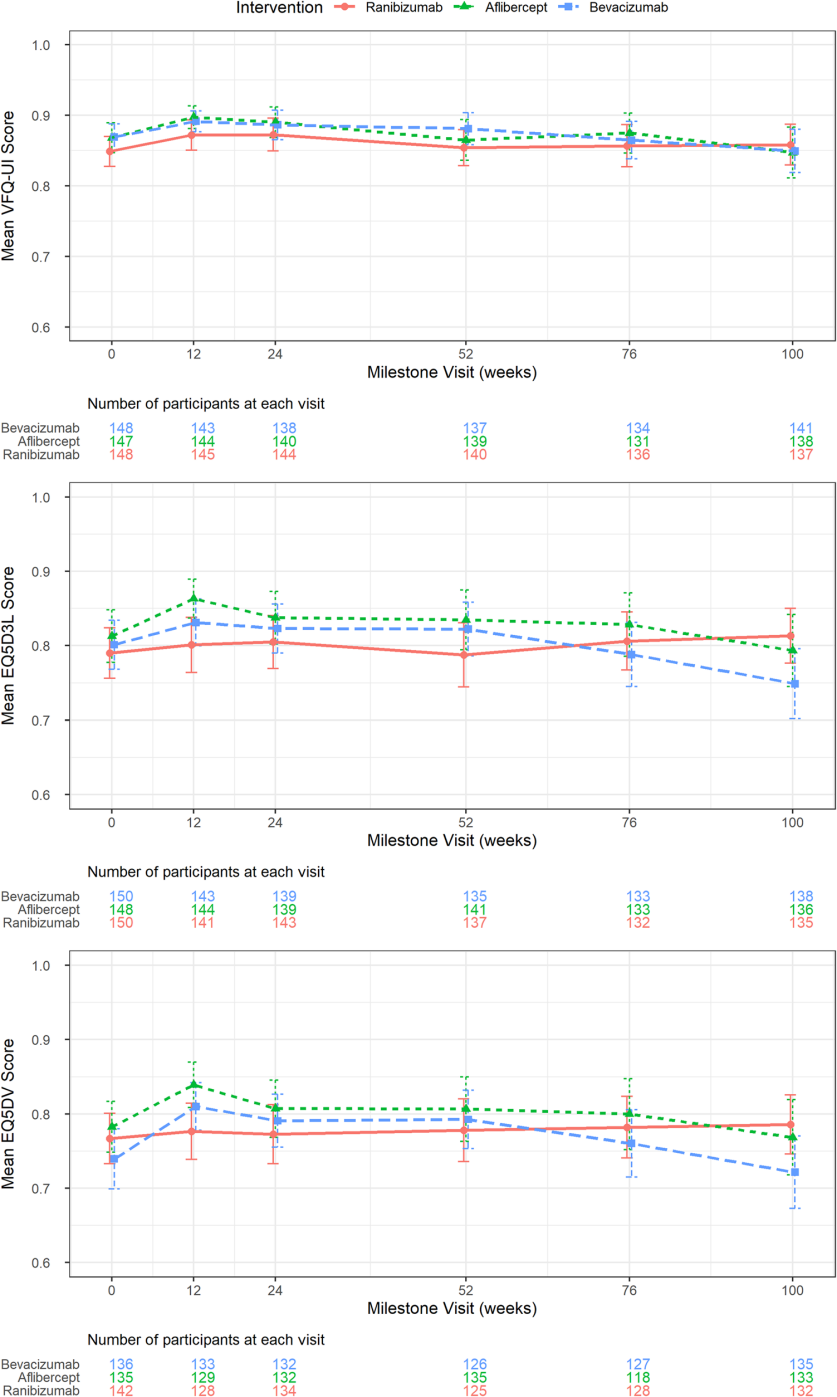
Utility scores over 100 weeks. *EQ5D3L* EQ-5D three level, *EQ5DV* EQ-5D with vision bolt-on, *VFQ*-*UI* Visual Functioning Questionnaire-Utility Index

Utility scores are shown in Fig. 2 with the number of participants in each arm providing data at each milestone visit summarised below each graph. Using VFQ-UI, bevacizumab led to the most QALYs (1.666) over the 100-week trial period, aflibercept the second most (1.651) and ranibizumab the least (1.627). There was no statistically significant difference in the QALYs for bevacizumab compared to ranibizumab and aflibercept. Bevacizumab dominated ranibizumab and aflibercept (ranibizumab and aflibercept were both more costly and less effective compared with bevacizumab). The probability that bevacizumab was the most cost-effective intervention compared to aflibercept and ranibizumab was 100% at £30,000 per QALY (Table 2).

#### Scenario Analyses

The complete case analysis and 52-week scenario using VFQ-UI led to the same findings as the base case. There was no statistically significant difference in QALYs (adjusted for the baseline utility score) between the three interventions, but bevacizumab was statistically significantly cheaper and thus dominated ranibizumab and aflibercept (Table 2).

Using EQ-5D and EQ-5D V, ranibizumab led to the fewest QALYs followed by bevacizumab and aflibercept, but the difference in QALYs was not statistically significant. In both cases, bevacizumab dominated ranibizumab, and the ICER for aflibercept vs ranibizumab was considerably above £30,000 per QALY (Table 2). Using a list price of £243 for bevacizumab, the total bevacizumab costs increased to £8933, but bevacizumab was still statistically significantly cheaper and continued to dominate ranibizumab and aflibercept (Table 2).

### Economic Model

#### Base Case

The base-case economic model analysis considered the long-term cost effectiveness of the treatments beyond the end of the LEAVO trial (mean 15.6 years). Bevacizumab was the least costly intervention (£18,353), followed by ranibizumab (£30,226) and aflibercept (£35,026). Bevacizumab was statistically significantly cheaper than ranibizumab and aflibercept (Table 3).

**Table 3:** Model-based analysis: base-case and scenario analysis results

	Total (95% CI)		Incremental (95% CI)^a^		ICER (£)
	Costs (£)	QALYs	Costs (£)	QALYs	QALYs
*Base-case analysis*					
Bevacizumab	18,353 (17,782–18,925)	9.678 (9.572–9.785)			
Ranibizumab	30,226 (29,386–31,066)	9.635 (9.512–9.757)	11,873 (11,458–12,288)	− 0.044 (− 0.074 to − 0.013)	Dominated
Aflibercept	35,026 (33,990–36,062)	9.569 (9.429–9.710)	16,673 (16,036–17,310)	− 0.109 (− 0.161 to − 0.057)	Dominated
*Scenario analysis: EQ-5D for utilities*					
Bevacizumab	18,353 (17.782–18,925)	8.782 (8.740–8.823)			
Ranibizumab	30,226 (29,386–31,066)	8.795 (8.754–8.836)	11,873 (11,458–12,288)	0.013 (0.008–0.018)	908,532
Aflibercept	35,026 (33,990–36,062)	8.832 (8.790–8.874)	4800 (4445–5154) vs ranibizumab	0.037 (0.032–0.043) vs ranibizumab	128,513 vs ranibizumab 330,697 vs bevacizumab
*Scenario analysis: EQ-5D V for utilities*					
Bevacizumab	18,353 (17.782–18,925)	8.346 (8.282–8.410)			
Ranibizumab	30,226 (29,386–31,066)	8.351 (8.283–8.419)	11,873 (11,458–12,288)	0.005 (− 0.007 to 0.017)	2,491,676
Aflibercept	35,026 (33,990–36,062)	8.369 (8.289–8.449)	4800 (4445–5154) vs ranibizumab	0.018 (0.000–0.045) vs ranibizumab	268,963 vs ranibizumab 737,383 vs bevacizumab
*Scenario analysis: 100-week time horizon*					
Bevacizumab	6349 (6293–6405)	1.641 (1.631–1.651)			
Ranibizumab	15,254 (14,962–15,545)	1.641 (1.631–1.651)	8905 (8650–9161)	0.000 (− 0.000 to 0.001)	34,067,841
Aflibercept	18,844 (18,438–19,249)	1.646 (1.636–1.655)	3590 (3400–3780)	0.005 (0.004–0.005)	793,348 (vs ranibizumab) 2,610,554 (vs bevacizumab)
*Scenario analysis: bevacizumab list price from BNF (£243)*					
Bevacizumab	23,530 (22,884–24,176)	9.678 (9.572–9.785)			
Ranibizumab	30,226 (29,386–31,066)	9.635 (9.512–9.757)	6696 (6400–6992)	− 0.044 (− 0.074 to − 0.013)	Dominated
Aflibercept	35,026 (33,990–36,062)	9.569 (9.429–9.710)	11,496 (10,961–12,030)	− 0.109 (− 0.161 to − 0.057)	Dominated

*BNF* British National Formulary, *CI* confidence interval, *EQ*-*5D* EuroQol-5 Dimension, *EQ*-*5D V* EuroQol-5 Dimension with Vision bolt-on, *ICER* incremental cost-effectiveness ratio, *QALY* quality-adjusted life-year

^a^Adjusted for baseline utility score

Using VFQ-UI, bevacizumab provided the most QALYs (9.678), followed by ranibizumab (9.635), and aflibercept (9.569)—equivalent to 0.5 and 1.3 additional months at full health. Bevacizumab led to statistically significantly more QALYs. Therefore, bevacizumab dominated ranibizumab and aflibercept. The probability that bevacizumab was the most cost-effective intervention was 98.6% at £20,000 per QALY.

#### Scenario Analyses

Using EQ-5D, bevacizumab provided the fewest QALYs followed by ranibizumab and aflibercept (difference equivalent to less than 1 month at full health). We compared ranibizumab to bevacizumab, and aflibercept to ranibizumab because an incremental analysis compares each intervention to the next most effective (excluding dominated interventions). The 95% CIs for the incremental QALYs for ranibizumab vs bevacizumab and aflibercept vs ranibizumab contained only positive values. Using EQ-5D V, the results suggested the same numerical trend as EQ-5D, but ranibizumab was not statistically significantly better. For both EQ-5D and EQ-5D V, ranibizumab was extendedly dominated (dominated by a combination of aflibercept and bevacizumab) and the ICERs for aflibercept vs bevacizumab were over £30,000 per QALY. The probability that bevacizumab was the most cost-effective intervention was 99.7% (EQ-5D) and 98.0% (EQ-5D V) at £20,000 per QALY and 99.0% (EQ-5D) and 97.1% (EQ-5D V) at £30,000 per QALY.

Using a list price of £243 for bevacizumab, the total costs for bevacizumab increased to £23,530 (95% CI £22,884–£24,176). In this scenario, the incremental costs for ranibizumab vs bevacizumab decreased, but bevacizumab continued to dominate both ranibizumab and aflibercept. The probability that bevacizumab was the most cost-effective intervention was 94.7% at £20,000 per QALY and 91.3% at £30,000 per QALY (Table 3).

#### 100-Week Time Horizon

Using a 100-week time horizon, bevacizumab and ranibizumab generated almost the same number of QALYs (1.641), and aflibercept generated slightly more QALYs (1.646) (Table 3). Bevacizumab remained the least costly intervention (£6349), followed by ranibizumab (£15,254) and aflibercept (£18,844). Ranibizumab was extendedly dominated and the ICER for aflibercept vs bevacizumab was above £30,000 per QALY and bevacizumab had 100% probability of being the most cost-effective intervention at £20,000 and £30,000 per QALY.

#### Disaggregated Costs

The cost breakdown per each intervention is shown in Table 4. To generate comparable intervention costs, assuming a cost of £28 per bevacizumab injection would require a discount of at least 95% on the list price of aflibercept and ranibizumab. Whether this would lead them to be cost effective at £30,000 per QALY would depend on the utility measure used. If bevacizumab were assumed to cost £243 per injection, aflibercept and ranibizumab would have comparable costs with a discount of 63% and 53% applied to the list price respectively.

**Table 4:** Model-based analysis: disaggregated costs (base case)

Costs, £ (95% CI)	Ranibizumab	Aflibercept	Bevacizumab
1. Treatment costs			
a. Study eye intervention costs	11,785 (11,387–12,184)	17,156 (16,582–17,730)	634 (614–654)
b. Study eye central subfield thickness examination and visit costs	5427 (5351–5503)	5372 (5299–5444)	5622 (5542–5701)
c. Non-study eye drug costs	771 (750–792)	1051 (1021–1081)	40 (39–41)
d. Non-study eye central subfield thickness examination and visit costs	268 (262–274)	249 (242–255)	276 (270–282)
2. Disease management costs	9588 (9049–10,127)	10,058 (9435–10,681)	9283 (8807–9759)
3. Ocular adverse event costs	1322 (1238–1405)	109 (101–117)	1392 (1301–1483)
4. Blindness costs	1065 (918–1212)	1031 (886–1176)	1107 (957–1257)
Total costs	30,226 (29,386–31,066)	35,026 (33,990–36,062)	18,353 (17,782–18,925)

## Discussion

### Principal Findings

Based on our assumptions and models, we found that bevacizumab was the most cost-effective intervention for the treatment of MO due to CRVO at £30,000 per QALY. Our findings were consistent between the model-based and within-trial analyses and robust to scenario analyses using alternative assumptions. This finding would change only if substantial discounts were offered on the price of ranibizumab or aflibercept. The inclusion of three utility measures in LEAVO allowed us to consider scenarios using vision-specific or generic measures of health. We found that the VFQ-UI led to more total QALYs for each intervention but the incremental QALYs were similar using the three measures.

We found that bevacizumab and ranibizumab did not generate significantly different QALYs; however, LEAVO found that bevacizumab was not non-inferior to ranibizumab when analysing the change in BCVA in the study eye [18]. This difference between QALY and visual acuity outcomes may be because patients’ overall sight is determined by their visual acuity in both eyes (the better and worse seeing eyes and the interaction between them), and thus HRQoL may not closely relate to assessment of visual acuity in one eye alone. Treatment becomes a difficult issue as clinicians and patients typically wish to optimise visual acuity in the affected eye, although this may not significantly alter the patient’s overall health and quality of life. Furthermore, treatment may change which eye is the better-seeing eye (if the worse-seeing eye is treated and improves to an extent that it is better than the original better-seeing eye), which will affect estimated utility in our models as the mappings use different coefficients for the better-seeing eye and worse-seeing eye.

Our findings suggest that to maximise health within a fixed general NHS healthcare budget, NHS clinicians in England could consider using bevacizumab instead of aflibercept or ranibizumab to treat MO due to CRVO. Whether this would lead to a small increase or decrease in health depends on the utility measure used. Such decisions may be considered controversial, particularly where the willingness to accept health losses differs from the willingness to pay for health gains. If aflibercept and ranibizumab were new interventions for treating MO due to CRVO, and bevacizumab was established standard care, it would be highly unlikely that NICE would consider aflibercept and ranibizumab cost-effective interventions at their list prices. Treatment with bevacizumab saves £5560 per year compared with aflibercept or £4545 compared with ranibizumab (see the ESM). If the estimated 5700 people diagnosed with MO due to CRVO each year in England and Wales (Royal College of Ophthalmologists) were treated with bevacizumab instead of aflibercept, the NHS would save £31,692,000 within 1 year (£25,906,500 if treated with bevacizumab instead of ranibizumab). Other healthcare systems would also see cost savings, provided the cost per injection for bevacizumab is lower than aflibercept and ranibizumab. Wider arguments are made for and against the use of bevacizumab for ophthalmologic indications in the NHS [37, 38].

Our base-case analysis for the bevacizumab injection was based on separating the larger vial into prefilled syringes (as used in the LEAVO study), i.e. compounding, at a cost of £28. In clinical practice, if there are additional costs associated with the compounding, then the total costs of bevacizumab will increase. However, the scenario analysis found that even assuming a full vial of bevacizumab using the list price, bevacizumab was still significantly cheaper than and dominated ranibizumab and aflibercept.

It is possible that patients in clinical practice may receive fewer injections than in LEAVO. For example, the real-world LUMINOUS study found that the mean number of ranibizumab injections in patients with CRVO was 4.2 at month 12 and 5.6 at month 24 [39]. These are somewhat lower than the mean number of ranibizumab injections at similar time points in LEAVO: 8.1 at week 52 and 11.8 at week 100 [18]. Assuming a similar reduction in injection frequency for ranibizumab, aflibercept and bevacizumab, we would expect the total costs of each intervention to be lower and therefore the cost saving associated with bevacizumab to be lower. However, reducing visits and treatments may not necessarily be appropriate. Based on a careful second year follow-up and anti-vascular endothelial growth factor therapy if reinjection criteria were met, patients in LEAVO maintained initial visual acuity gains at 2 years. This was not the case in certain trials such as COPERNICUS [40] where in principle, a 3-monthly follow-up in the second year led to visual acuity losses, nor can it definitely be concluded in real-world data such as LUMINOUS, where the drop-out rate by 2 years in the treatment-naïve CRVO cohort was high [39], making interpretation of visual acuity outcomes difficult. Therefore, care must be taken to ensure that reducing visit and injection frequency does not compromise visual acuity outcomes.

### Validation

The 100-week scenario analysis in the model gives similar results to the within-trial analysis, suggesting that the long-term model is valid for estimating short-term costs and QALYs. To validate the extrapolation, we compared our results to models for aflibercept and ranibizumab in CRVO. Evidence submitted by Novartis for NICE’s appraisal of ranibizumab considered a 15-year time horizon and reported lower mean survival (12.3 years), QALYs (7.55 using VFQ-UI) and costs (£26,327) than our model, as would be expected for a shorter time horizon [41]. Most of the results of Bayer’s cost-effectiveness analysis for NICE’s appraisal of aflibercept are redacted, but the QALY gain for aflibercept vs ranibizumab over a lifetime was reported to be 0.054 using EQ-5D [42]—our model gave results of a similar magnitude.

A recent systematic review of the three interventions across other retinal conditions identified two large US trials that provided evidence that ranibizumab and aflibercept are not cost effective compared to bevacizumab in other retinal conditions [43]. Additionally, a large UK-based trial in age-related macular degeneration was inconclusive in comparing bevacizumab to ranibizumab using BCVA [44]. The trial found that ranibizumab is not cost effective compared to bevacizumab owing to its substantially larger costs and small QALY gain [45]. The cost-effectiveness results for MO in our study are consistent with these findings.

Our assumption that LEAVO is representative of the patient population in England appears valid when considering that LEAVO was conducted entirely in UK settings, and when comparing against the international real-world LUMINOUS study, which had a similar baseline age (LEAVO: 69.1, LUMINOUS: 69.7), proportion of female patients (LEAVO: 48.8, LUMINOUS: 41.5) and baseline BCVA (LEAVO: 54.1, LUMINOUS: 44.7) [18, 39]. Although baseline BCVA is higher in LEAVO than LUMINOUS, a prespecified subgroup analysis in LEAVO found no statistically significant differences across subgroups in baseline BCVA score for changes in BCVA letter scores from baseline to 100 weeks [18].

### Strengths and Limitations

Our study used a within-trial and model-based analysis to estimate the cost effectiveness of the three interventions, with the same findings. Both methods followed best practice guidelines [20, 21, 24, 46]. We avoided arbitrarily categorising BCVA scores into health states, thus we were able to model small changes in visual acuity, and incorporated heterogeneity by modelling patients with different baseline characteristics while preserving the relationship between different characteristics. We were able to model the study and non-study eye separately and use both to predict utility and resource use.

Using patient-level data from LEAVO enabled us to predict BCVA and CST change that accounted for patient-level characteristics as well as trends over time. Our mappings used flexible models to account for the unique distributions of utility data. While LEAVO provided rich data for the study period, health economics data (comparable by arm) were missing. Resource use questionnaires are also vulnerable to recall bias. In LEAVO, resource use marginally contributed to the overall total cost in each arm; therefore, it is unlikely to change the health economic conclusions. Additionally, the multiple imputation model suggested that the results were consistent with those from the complete case analysis.

LEAVO was limited to a duration of 100 weeks and thus did not provide long-term data on the effectiveness or safety. We identified limited long-term data in a systematic review that we could use to populate the model. This led to uncertainty in the long-term extrapolations of effectiveness, withdrawals, adverse events and development of MO in the non-study eye. For natural history, we relied on a study of the general population from over 20 years ago (Beaver Dam Study) as newer studies did not provide numerical data [47].

## Conclusions

Although LEAVO could not demonstrate bevacizumab to be non-inferior to ranibizumab and aflibercept in terms of visual acuity gain, our analysis suggests that bevacizumab was the most cost-effective intervention, in terms of cost per QALY using VFQ-UI or EQ-5D, for the treatment of MO due to CRVO. While patients, funders and ophthalmologists should be fully informed about the clinical efficacy of bevacizumab compared to aflibercept or ranibizumab, its routine use for MO secondary to CRVO would lead to substantial cost savings. Whether this would lead to small health gains or losses depends on the outcome measure used to determine HRQoL.

## Supplementary Information

Below is the link to the electronic supplementary material.

Supplementary file 1 (DOCX 174 kb)

## Declarations

### Availability of Data and Material

Summary-level data are available from the LEAVO study. Model input data are provided in the article and Appendix in the ESM. Patient-level data from LEAVO are not publicly available.
