# Correction to: Cost Effectiveness of Ranibizumab vs Aflibercept vs Bevacizumab for the Treatment of Macular Oedema Due to Central Retinal Vein Occlusion: The LEAVO Study

**DOI:** 10.1007/s40273-021-01057-y

**Published:** 2021-06-19

**Authors:** Becky Pennington, Abualbishr Alshreef, Laura Flight, Andrew Metry, Edith Poku, Philip Hykin, Sobha Sivaprasad, A. Toby Prevost, Joana C. Vasconcelos, Caroline Murphy, Joanna Kelly, Yit Yang, Andrew Lotery, Michael Williams, John Brazier

**Affiliations:** 1 School of Health and Related Research, University of Sheffield, Sheffield, UK; 2 NIHR Moorfields Biomedical Research Centre, London, UK; 3 Nightingale-Saunders Clinical Trials and Epidemiology Unit at King’s Clinical Trials Unit, King’s College London, London, UK; 4 King’s Clinical Trials Unit at King’s Health Partners, King’s College London, London, UK; 5 Wolverhampton Eye Infirmary, Wolverhampton, UK; 6 Faculty of Medicine, University of Southampton, Southampton, UK; 7 Centre for Medical Education, Queen’s University of Belfast, Belfast, UK

## Correction to: PharmacoEconomics 10.1007/s40273-021-01026-5

The article Cost Effectiveness of Ranibizumab vs Aflibercept vs Bevacizumab for the Treatment of Macular Oedema Due to Central Retinal Vein Occlusion: The LEAVO Study,written by Becky Pennington, Abualbishr Alshreef, Laura Flight, Andrew Metry, Edith Poku, Philip Hykin, Sobha Sivaprasad, A. Toby Prevost , Joana C. Vasconcelos, Caroline Murphy, Joanna Kelly , Yit Yang, Andrew Lotery, Michael Williams and John Brazier was published under the incorrect Creative Commons (CC) license (CC-BY-NC). The correct license is CC-BY. Open access for this paper was funded by the NIHR HTA programme through Moorfields Eye Hospital.

This article is licensed under a Creative Commons Attribution 4.0 International License, which permits use, sharing, adaptation, distribution and reproduction in any medium or format, as long as you give appropriate credit to the original author(s) and the source, provide a link to the Creative Commons license, and indicate if changes were made. The images or other third-party material in this article are included in the article’s Creative Commons license, unless indicated otherwise in a credit line to the material. If material is not included in the article’s Creative Commons license and your intended use is not permitted by statutory regulation or exceeds the permitted use, you will need to obtain permission directly from the copyright holder. To view a copy of this license, visit https://creativecommons.org/licen ses/by/4.0/.

The original article has been corrected.

