# Supplementary material for: Cost Effectiveness of Ranibizumab vs Aflibercept vs Bevacizumab for the Treatment of Macular Oedema Due to Central Retinal Vein Occlusion: The LEAVO Study
Source: Pharmacoeconomics. 2021 Apr 26;39(8):913–27. doi: 10.1007/s40273-021-01026-5 (PMC8298346; doi:10.1007/s40273-021-01026-5)
Supplement: Supplementary file 1 — (DOCX 174 kb) [file 40273_2021_1026_MOESM1_ESM.docx]

**Supplementary material**

1. **Modelling withdrawal**

Survival analysis was used to fit parametric models to extrapolate time to withdrawal beyond the trial period, following good practice guidance (1). The three interventions are modelled separately to reflect numerical differences, despite non-statistically significant differences in the data (p=0.572). AIC and BIC are similar between parametric models, and no external validation was possible due to a lack of data. The Weibull distribution is used to model time to withdrawal event.

1. **Modelling ocular adverse events**

Survival analysis was used to fit parametric models to extrapolate time to event beyond the trial period, following good practice guidance (1). The log-rank test found no statistically significant difference between the time to first adverse event and time to subsequent adverse events (p=0.128), and the number of subsequent adverse events was small, so the time to first adverse event is used as the time to first or subsequent adverse events in the model.

Although the time to adverse event is not statistically significantly different between the interventions (p=0.683), they are modelled separately to reflect numerical differences in the deterministic analysis. The probabilistic analysis considers the uncertainty around the point estimates reflecting that the interventions are not significantly different. According to the Akaike Information Criterion (AIC) and Bayesian Information Criterion (BIC), the Weibull was the best fitting parametric model. As no data were available on the ocular adverse event rates for any of the three interventions beyond the trial period (see *Chapter 4.3*), external validation is not possible. The Weibull is therefore used to model the time to adverse events.

As the model considers any ocular adverse event, modelled patients who experience ocular adverse events incur the average cost for an ocular adverse event, based on the proportion of patients in LEAVO experiencing each ocular adverse event. This is calculated by multiplying the number of each type of ocular adverse event by the cost for treating that ocular adverse event, and dividing the total by the number of patients experiencing ocular adverse events in LEAVO. The cost per ocular adverse event is the same for the three interventions, £317.96. Costs for each ocular adverse event are from NHS Reference Costs^112^ or the British National Formulary,^111^.

1. **Modelling BCVA and CST change over time**

The retreatment algorithm assesses both OCT CST and BCVA, so both must be modelled for treated eyes. BCVA in both eyes is important for predicting HRQoL, so BCVA is modelled for both eyes.

##### *Treated eyes*

Growth models (longitudinal analyses to estimate growth trajectories over a period of time) are fitted to CST and BCVA from the LEAVO trial data. In these models, CST (or BCVA) at weeks 12, 24, 52, 76 and 100 are estimated as a function of time, baseline CST (or BCVA), age at baseline, intervention, number of injections and time since last injection. Gender is found not be a significant predictor of CST or BCVA, so is excluded. Intervention is not a significant predictor of CST or BCVA, but is included to reflect numerical differences between the interventions.

The equation for $y_{it}$, the BCVA score for patient $i$ at time $t$ is:

$y_{it}= \eta_{1i}+ \eta_{2i}\times t+\gamma_{1t}\times number of injections+ \gamma_{2t}\times days since injection+ \varepsilon_{it}$ (1)

Where

$\eta_{1i}=+ \eta_{1}+\alpha_{1}\times\frac{age at baseline}{10}+\alpha_{2} \times\frac{BCVA at baseline}{10}+ \alpha_{3} \times tn2+ \alpha_{4} \times tn3+\xi_{i}^{1}$ (2)

And

$\eta_{2i}=+ \eta_{2}+\beta_{1} \times\frac{age at baseline}{10}+\beta_{2} \times\frac{BCVA at baseline}{10}+ \beta_{3} \times tn2+ \beta_{4} \times tn3+\xi_{i}^{2}$ (3)

Where $tn2$ = 1 for aflibercept and 0 otherwise, and $tn3=1$ for bevacizumab and 0 otherwise, and $\xi$ is an error term.

(The equation for CST follows the same structure, but uses CST at baseline/100 instead of BCVA at baseline/10).

Whereas $\eta, \alpha, \beta$ (age at baseline, CST or BCVA at baseline and intervention) are time-invariant covariates, $\gamma_{1}$and $\gamma_{2}$(number of injections and time since last injection) are time-variant covariates, with values only available at 12, 24, 52 and 76 weeks. To estimate CST and BCVA in the economic model, these covariates are used at week 12, 24, 52, 76 and 100 visits. For other visits, the following approach is used:

*Weeks 4 and 8:* CST and BCVA are calculated at week 12, and linear interpolation is used to estimate CST and BCVA at week 4 and week 8 visits.

*Visits from 16 to 100, excluding weeks 24, 52:* CST and BCVA are calculated for the closest milestone visits before and after the non-milestone visits, and interpolation is used to estimate BCVA at the non-milestone visits.

*Visits beyond week 76:* The time-varying covariates appear similar towards the end of LEAVO, and so models which restricted these covariates to be the same at week 76 and 100 were compared to unrestricted models. Log-likelihood tests indicated that the null hypothesis that the restricted models were true should not be rejected. The restricted models suggest that the effect of the number of injections and time since last injection flatten towards the end of LEAVO and can therefore be used to extrapolate beyond 100 weeks.

***Untreated eyes***

Untreated eyes are considered to be eyes that never received treatment in LEAVO and eyes from which treatment has ended or been withdrawn. The same assumption is used for treated eyes where the most recent injection was at least one year ago.

CST is not modelled for the non-study eye, unless the patient develops ME in the non-study eye. In this case, CST and BCVA for the non-study eye are modelled using the same approach as the study eye.

BCVA is modelled for untreated eyes using natural history data. The Beaver Dam study was a large population-based study that recorded BCVA in patients over five years This study reports^93^ the letters gained or lost in the left and right eye for people aged under 55, 56-65, 65-74 and 75 and over and has been used in previous CRVO economic models.^50^ Combining the right and left eye data, the annual average decrease in BCVA is -0.02 (standard error (SE): 0.04) for ages 55-64, 0.26 (SE: 0.04) for ages 65-74, and 0.76 (SE: 0.06) for ages 75+. There is no change for people aged less than 55. These data appear consistent with a study of the natural history in CRVO, which reports that increasing age was positively associated with visual acuity deterioration, and over two to five years, in eyes with non-ischaemic CRVO ME, 14% improved, 47% stayed the same, and 39% worsened.^94^

1. **52 week time horizon from within trial**

Within-trial analysis: Scenario analysis using 52 week time horizon

|  | **Outcome** | **Intervention**  **Mean (SD); N** | **Comparator**  **Mean (SD); N** | **Difference**  **Mean (95% CI)^a^** | **Probability CE £20000 (£30000)** |
| --- | --- | --- | --- | --- | --- |
| **Aflibercept**  **vs Ranibizumab** | **Cost (£)** | 9,214 (2,235);154 | 8,164 (2,163);154 | 1,002 (516 to 1,487) | - |
|  | **QALY** | 0.8798 (0.1208);154 | 0.865 (0.1230);154 | 0.004 (-0.0178 to 0.0256) | - |
|  | **ICER (£)** | | | 256,547 | 0.00 (0.02) |
| **Bevacizumab vs Ranibizumab** | **Cost (£)** | 3,621 (2,017);154 | 8,164 (2,163);154 | -4,546 (-4,999 to -4,093) | - |
|  | **QALY** | 0.8842 (0.1171); 154 | 0.865 (0.1230);154 | 0.007 (-0.0143 to 0.0290) | - |
|  | **ICER (£)** | | | Bevacizumab is dominant | 1.00 (1.00) |
| **Aflibercept vs Bevacizumab** | **Cost (£)** | 9,214 (2,235);154 | 3,621 (2,017);154 | 5,560 (5,082 to 6,039) | - |
|  | **QALY** | 0.8798 (0.1208);154 | 0.8842 (0.1171); 154 | -0.004(-0.0256 to 0.0168) | - |
|  | **ICER (£)** | | | Aflibercept is dominated | 0.00 (0.00) |

CI; confidence interval, ICER; incremental cost-effectiveness ratio, QALY; quality adjusted life year, SD; standard deviation **^a^** adjusted for baseline utility score

1. Within-trial analysis: Disaggregated costs

| **Cost per patient (£)** | **Ranibizumab**  **Mean (SD); N** | **Aflibercept**  **Mean (SD); N** | **Bevacizumab**  **Mean (SD); N** | **Aflibercept vs Ranibizumab**  **Mean (95% CI)** | **Bevacizumab vs Ranibizumab**  **Mean (95% CI)** | **Aflibercept vs Bevacizumab**  **Mean (95% CI)** |
| --- | --- | --- | --- | --- | --- | --- |
| **Blindness** | 1.94  (15.28);125 | 4.70  (23.51);129 | 2.96  (18.79);123 | 2.76  (-2.05 to 7.57) | 1.02  (-3.85 to 5.88) | -1.74  (-6.57 to 3.08) |
| **Concomitant Medications** | 69.03  (342.27);154 | 22.86  (26.40);154 | 124.37  (907.96);154 | -46.17  (-171.35 to 79.01) | 55.34  (-69.84 to 180.52) | 101.51(  -23.67 to 226.69) |
| **Concomitant Procedures** | 173.23  (567.30);154 | 222.60  (749.14);154 | 217.57  (880.10);154 | 49.37  (-116.66,215.4) | 44.34  (-121.69,210.37) | -5.03  (-171.06,161) |
| **Continuous Care and Support** | 7.11  (54.99);99 | 38.76  (172.27);88 | 10.43  (82.93);90 | 31.66  (-0.75,64.07) | 3.32  (-28.89,35.54) | -28.33  (-61.5,4.83) |
| **Health Care Contacts** | 729.36  (815.88);91 | 710.46  (920.25);92 | 740.14  (1,065.62);81 | -18.89  (-289.62 to 251.84) | 10.78  (-268.94 to 290.51) | 29.68  (-249.33 to 308.68) |
| **Hospital Admissions** | 54.17  (479.35);149 | 34.08  (239.58);149 | 89.32  (689.04);148 | -20.10  (-134.43,94.23) | 35.15  (-79.37,149.67) | -55.24  (-169.76,59.28) |
| **Intervention** | 10,991.74  (3,973.57);154 | 12,445.31  (4,231.59);154 | 4,784.99  (1,247.34);154 | 1,453.57  (687.9 to 2,219.23) | -6,206.74  (-6,972.41 to -5,441.08) | 7660.31  (6,894.65 to8,425.98) |
| **Total Costs** | 13,014  (3,605); 154 | 14,328  (3,773);154 | 6292  (3371);154 | 1,245  (421 to 2,070) | -6,760  (-7,546 to-5,973) | 7,984  (7,209 to 8,759) |

CI; confidence interval, SD; standard deviation

1. **Model input parameters**

| **Parameter** | **Distribution** | **Mean (standard error)** | **Source (mean)** | **Source for standard error** | |
| --- | --- | --- | --- | --- | --- |
| **Intervention and related costs** | | | | | |
| Ranibizumab injection | N/A | £551.00 | BNF 2019(2) | N/A | |
| Aflibercept injection | N/A | £816.00 | BNF 2019(2) | N/A | |
| Bevacizumab injection | N/A | £28.00 | Judicial review (3) | N/A | |
| CST cost | Gamma | £108.21 | Department of Health (2018) ^(4)^  NHS codes BZ87A | Quartile data of the NHS codes  Department of Health (2017)(5) | |
| First visit cost | Gamma | £140.04 | Department of Health (2018) ^(4)^  NHS codes WF02B |  |  |
| Follow-up visit cost | Gamma | £105.19 | Department of Health (2018) ^(4)^  NHS codes WF02A |  |  |
| **Costs associated with resource use** | | | | | |
| A&E visit cost | Gamma | £160.23 (£9.34) | Department of Health (2018) ^(4)^  Weighted average for NHS codes VB01Z to VB11Z | Quartile data of the NHS codes (weighted)  Department of Health (2017)(5) | |
| Visit Cost of ocular A&E | Gamma | £118.02 (£2.67) | Department of Health (2018) ^(4)^  NHS codes WF01B | Quartile data of the NHS codes  Department of Health (2017)(5) | |
| Visit Cost of eye consultant | Gamma | £95.13 (£1.85) | Department of Health (2018) ^(4)^  NHS codes WF01A |  |  |
| Call cost to ophthalmologist | Gamma | £28.20 (£4) | Department of Health (2018) ^(4)^  NHS codes WF01D |  |  |
| Visit Cost of optometrist/optician | Gamma | £76.50 (£10.5) | Department of Health (2018) ^(4)^  NHS codes WF01B |  |  |
| Visit Cost for low vision appointment | N/A | £153.00 | Estimated to be double the visit cost of optometrist/optician | | |
| Visit Cost of GP | Gamma | £37.40 (£3.74) | Curtis and Burns (2018)(6) | 10% assumption around the mean | |
| Visit Cost of practice nurse | Gamma | £17.79 (£1.78) |  |  |  |
| Call cost to GP | Gamma | £28.00 (£2.8) |  |  |  |
| **Resource use parameters (3 monthly)** | | | | | |
| A&E visit: WSE | Multinormal | -0.001 | Analysis of LEAVO data | | |
| A&E visit: constant |  | 0.103 |  |  |  |
| Eye A&E visit: WSE | Multinormal | -0.002 |  |  |  |
| Eye A&E visit: constant |  | 0.183 |  |  |  |
| GP visit: WSE | Multinormal | -0.004 |  |  |  |
| GP visit: constant |  | 0.441 |  |  |  |
| GP call: WSE | Multinormal | -0.001 |  |  |  |
| GP call: constant |  | 0.082 |  |  |  |
| Eye consultant visit: WSE | Multinormal | -0.004 |  |  |  |
| Eye consultant visit: constant |  | 1.163 |  |  |  |
| Low vision appointment: WSE | Multinormal | -0.002 |  |  |  |
| Low vision appointment: constant |  | 0.137 |  |  |  |
| Nurse appointment: WSE | Multinormal | -0.001 |  |  |  |
| Nurse appointment: constant |  | 0.083 |  |  |  |
| Optometrist appointment: WSE | Multinormal | 0.000 |  |  |  |
| Optometrist: constant |  | 0.054 |  |  |  |
| Ophthalmologist call: mean | Normal | 0.013 (0.007) |  |  |  |
| Helpline call: mean | Normal | 0.025 (0.009) |  |  |  |
| **Blindness costs** | | | | | |
| Percentage requiring community care | Beta | 6% (0.6%) | Colquitt et al (2008)(7) | 10% assumption around mean | |
| Percentage requiring hip replacement | Beta | 5% (0.5%) | Colquitt et al (2008)(7) | 10% assumption around mean | |
| Percentage requiring low vision aids | Beta | 33% (0.05%) | Colquitt et al (2008)(7) | Margrain et al (1999) (8) | |
| Percentage requiring low vision rehabilitation | Beta | 11% (1.1%) | Colquitt et al (2008)(7) | 10% assumption around mean | |
| Percentage requiring residential care | Beta | 30% (3%) | Colquitt et al (2008 (7) | 10% assumption around mean | |
| Percentage requiring treatment for depression | Beta | 39% (5.8%) | Colquitt et al (2008)(7) | Galaria et al (2000)(9) | |
| Percentage requiring blindness registration | Beta | 95 % (0.05%) | Colquitt et al (2008)(7) | Owen et al (2003)(10) | |
| Cost of community care (annual) | Gamma | £10,060.95 (£1,006.10) | Curtis and Burns 2018 (6) | 10% assumption around mean | |
| Cost of hip replacement (annual) | Gamma | £4,170.00 (£417.00) | Department of Health (2018)(4)  Code HT14C | 10% assumption around mean | |
| Cost of low vision aids (one-off) | Gamma | £194.41 (£19.44) | Meads 2003(11), Curtis and Burns (2018)(6) | 10% assumption around mean | |
| Cost of low vision rehabilitation (one-off) | Gamma | £153 | Estimated to be double the visit cost of optometrist/optician | | |
| Cost of residential care (annual) | Gamma | £6,000.80 (£600.08) | Curtis and Burns 2018(6) | 10% assumption around mean | |
| Cost of treatment for depression (annual) | Gamma | £2,430.58 (£243.06) | NICE, 2017 (TA460)(12) | 10% assumption around mean | |
| Cost of blindness registration (one-off) | Gamma | £60.50 (£6.05) | Curtis and Burns 2018(6) | 10% assumption around mean | |
| **Adverse events** | | | | | |
| Cost of adverse event | Gamma | £317.96 (£2.58) | Department of Health (2018)(4) | Weighted variance from NHS reference costs | |
| Weibull distribution: shape parameter | Multinormal | 0.745 | Analysis of LEAVO data | | |
| Weibull distribution: scale parameter – constant |  | -2.271 |  |  |  |
| Weibull distribution: scale parameter – aflibercept |  | -0.271 |  |  |  |
| Weibull distribution: scale parameter – bevacizumab |  | -0.049 |  |  |  |
| **Withdrawal** | | | | | |
| Weibull distribution: shape parameter | Multinormal | 0.326 | Analysis of LEAVO data | | |
| Weibull distribution: scale parameter – constant |  | -2.966 |  |  |  |
| Weibull distribution: scale parameter – aflibercept |  | 0.126 |  |  |  |
| Weibull distribution: scale parameter – bevacizumab |  | -0.227 |  |  |  |
| **Mortality: hazard ratios for CRVO** | | | | | |
| Female: aged 0-49 | Lognormal | 0.83 (2.89) | Bertelsen et al (2013) (13) | Calculated from confidence intervals | |
| Female: aged 50-59 | Lognormal | 1.49 (1.86) | Bertelsen et al (2013) (13) | Calculated from confidence intervals | |
| Female: aged 60-69 | Lognormal | 1.94 (1.27) | Bertelsen et al (2013) (13) | Calculated from confidence intervals | |
| Female: aged 70-79 | Lognormal | 0.94 (1.25) | Bertelsen et al (2013) (13) | Calculated from confidence intervals | |
| Female: aged 80 and over | Lognormal | 1.04 (1.23) | Bertelsen et al (2013) (13) | Calculated from confidence intervals | |
| Male: aged 0-49 | Lognormal | 1.49 (1.88) | Bertelsen et al (2013) (13) | Calculated from confidence intervals | |
| Male: aged 50-59 | Lognormal | 1.71 (1.54) | Bertelsen et al (2013) (13) | Calculated from confidence intervals | |
| Male: aged 60-69 | Lognormal | 1.17 (1.3) | Bertelsen et al (2013) (13) | Calculated from confidence intervals | |
| Male: aged 70-79 | Lognormal | 1.24 (1.14) | Bertelsen et al (2013) (13) | Calculated from confidence intervals | |
| Male: aged 80 and over | Lognormal | 1.26 (1.22) | Bertelsen et al (2013) (13) | Calculated from confidence intervals | |
| **BCVA and CST modelling** | | | | | |
| BCVA: baseline age/10 on intercept | Normal | -0.19728 (0.049) | Analysis of LEAVO data | | |
| BCVA: baseline BCVA/10 on intercept | Normal | 0.56235 (0.041) |  |  |  |
| BCVA: aflibercept on intercept | Normal | 0.18927 (0.155) |  |  |  |
| BCVA: bevacizumab on intercept | Normal | 0.03001 (0.154) |  |  |  |
| BCVA: baseline age/10 on slope | Normal | -0.25323 (0.06) |  |  |  |
| BCVA: baseline BCVA/10 on slope | Normal | -0.15787 (0.047) |  |  |  |
| BCVA: aflibercept on slope | Normal | -0.04577 (0.186) |  |  |  |
| BCVA: bevacizumab on slope | Normal | -0.06674 (0.18) |  |  |  |
| BCVA: days since injection at 12 weeks | Normal | -0.00083 (0.005) |  |  |  |
| BCVA: days since injection at 24 weeks | Normal | -0.00536 (0.001) |  |  |  |
| BCVA: days since injection at 52 weeks | Normal | 0.00069 (0.001) |  |  |  |
| BCVA: days since injection at 76+ weeks | Normal | -0.00026 (0.0001) |  |  |  |
| BCVA: number of injection at 12 weeks | Normal | 0.10891 (0.072) |  |  |  |
| BCVA: number of injection at 24 weeks | Normal | 0.06345 (0.035) |  |  |  |
| BCVA: number of injection at 52 weeks | Normal | -0.00871 (0.021) |  |  |  |
| BCVA: number of injection at 76+ weeks | Normal | -0.01121 (0.019) |  |  |  |
| BCVA: intercept | Multinormal | 4.811 |  |  |  |
| BCVA: slope | Multinormal | 2.878 |  |  |  |
| CST: baseline age/10 on intercept | Normal | -0.1953 (0.048) |  |  |  |
| CST: baseline CST/10 on intercept | Normal | 0.13111 (0.029) |  |  |  |
| CST: aflibercept on intercept | Normal | -0.46501 (0.151) |  |  |  |
| CST: bevacizumab on intercept | Normal | 0.22923 (0.149) |  |  |  |
| CST: baseline age/10 on slope | Normal | 0.29301 (0.067) |  |  |  |
| CST: baseline CST/10 on slope | Normal | -0.04915 (0.039) |  |  |  |
| CST: aflibercept on slope | Normal | 0.36749 (0.205) |  |  |  |
| CST: bevacizumab on slope | Normal | -0.02506 (0.197) |  |  |  |
| CST: days since injection at 12 weeks | Normal | 0.00231 (0.007) |  |  |  |
| CST: days since injection at 24 weeks | Normal | 0.02045 (0.003) |  |  |  |
| CST: days since injection at 52 weeks | Normal | 0.00239 (0.001) |  |  |  |
| CST: days since injection at 76+ weeks | Normal | 0.00144 (0.001) |  |  |  |
| CST: number of injection at 12 weeks | Normal | -0.00612 (0.103) |  |  |  |
| CST: number of injection at 24 weeks | Normal | -0.0594 (0.056) |  |  |  |
| CST: number of injection at 52 weeks | Normal | 0.06798 (0.027) |  |  |  |
| CST: number of injection at 76+ weeks | Normal | 0.06327 (0.022) |  |  |  |
| CST: intercept | Multinormal | 3.76348 |  |  |  |
| CST: slope | Multinormal | -2.75221 |  |  |  |
| **Annual BCVA change** | | | | | |
| Age 55-64: mean | Normal | 0.0200 (0.002) | Klein et al (1996)(14) | | 10% assumption around mean |
| Age 55-64: standard deviation | Normal | 0.0400 (0.004) | Klein et al (1996) (14) | | 10% assumption around mean |
| Age 65-74: mean | Normal | -0.2600 (0.026) | Klein et al (1996) (14) | | 10% assumption around mean |
| Age 65-74: standard deviation | Normal | 0.0400 (0.004) | Klein et al (1996) (14) | | 10% assumption around mean |
| Age 65-74: mean | Normal | -0.7600 (0.076) | Klein et al (1996) (14) | | 10% assumption around mean |
| Age 65-74: standard deviation | Normal | 0.0602 (0.060) | Klein et al (1996) (14) | | 10% assumption around mean |
| **Utility parameters: VFQ-UI** | | | | | |
| Component 1: BSE/10 | Multinormal | -0.00025 | Analysis of LEAVO | | |
| Component 1: WSE/10 |  | -0.00033 |  |  |  |
| Component 1: Age/10 |  | 0.00922 |  |  |  |
| Component 1: Male |  | 0.00110 |  |  |  |
| Component 1: Constant |  | 0.88490 |  |  |  |
| Component 2: BSE/10 |  | 0.02353 |  |  |  |
| Component 2: WSE/10 |  | 0.01637 |  |  |  |
| Component 2: Age/10 |  | 0.03448 |  |  |  |
| Component 2: Male |  | 0.00751 |  |  |  |
| Component 2: Constant |  | 0.18926 |  |  |  |
| Component 3: BSE/10 |  | 0.00372 |  |  |  |
| Component 3: WSE/10 |  | -0.00187 |  |  |  |
| Component 3: Age/10 |  | 0.00638 |  |  |  |
| Component 3: Male |  | -0.00413 |  |  |  |
| Component 3: Constant |  | 0.83403 |  |  |  |
| Probability of component 1 membership: BSE/10 |  | 0.25197 |  |  |  |
| Probability of component 1 membership: WSE/10 |  | 0.23102 |  |  |  |
| Probability of component 1 membership: Constant |  | -2.31366 |  |  |  |
| Probability of component 2 membership: BSE/10 |  | -0.41024 |  |  |  |
| Probability of component 2 membership: WSE/10 |  | -0.04126 |  |  |  |
| Probability of component 2 membership: Constant |  | 4.00996 |  |  |  |
| Component 1: log sigma |  | -4.78402 |  |  |  |
| Component 2: log sigma |  | -2.24672 |  |  |  |
| Component 3: log sigma |  | -3.49052 |  |  |  |
| **Utility parameters: EQ-5D** | | | | | |
| Component 1: BSE/10 | Multinormal | 0.01626 | Analysis of LEAVO | | |
| Component 1: WSE/10 |  | 0.01022 |  |  |  |
| Component 1: Age/10 |  | -0.02851 |  |  |  |
| Component 1: Male |  | 0.02663 |  |  |  |
| Component 1: Constant |  | 0.86003 |  |  |  |
| Component 2: BSE/10 |  | 0.01693 |  |  |  |
| Component 2: WSE/10 |  | -0.02069 |  |  |  |
| Component 2: Age/10 |  | 0.04236 |  |  |  |
| Component 2: Male |  | 0.20485 |  |  |  |
| Component 2: Constant |  | 0.01774 |  |  |  |
| Probability of component 1 membership: BSE/10 |  | 0.39593 |  |  |  |
| Probability of component 1 membership: WSE/10 |  | 0.24805 |  |  |  |
| Probability of component 1 membership: Constant |  | -2.76469 |  |  |  |
| Component 1: log sigma |  | -1.99075 |  |  |  |
| Component 2: log sigma |  | -1.32132 |  |  |  |
| **Utility parameters: EQ-5D V** | | | | | |
| Component 1: BSE/10 | Multinormal | 0.00378 | Analysis of LEAVO | | |
| Component 1: WSE/10 |  | -0.00730 |  |  |  |
| Component 1: Age/10 |  | 0.04348 |  |  |  |
| Component 1: Male |  | 0.20676 |  |  |  |
| Component 1: Constant |  | 0.03574 |  |  |  |
| Component 2: BSE/10 |  | 0.02012 |  |  |  |
| Component 2: WSE/10 |  | 0.01255 |  |  |  |
| Component 2: Age/10 |  | -0.01937 |  |  |  |
| Component 2: Male |  | 0.01592 |  |  |  |
| Component 2: Constant |  | 0.73587 |  |  |  |
| Probability of component 1 membership: BSE/10 |  | -0.53561 |  |  |  |
| Probability of component 1 membership: WSE/10 |  | -0.20177 |  |  |  |
| Probability of component 1 membership: Constant |  | 3.77924 |  |  |  |
| Component 1: log sigma |  | -1.25309 |  |  |  |
| Component 2: log sigma |  | -1.93060 |  |  |  |
| A&E, Accident and Emergency; BCVA, best corrected visual acuity; BSE, Better Seeing Eye; CST, central subfield thickness; EQ-5D, EuroQol-Five Dimension; EQ-5D V, EQ-5D with vision bolt-on; GP, General Practitioner; N/A, Not applicable; VFQ-UI, Visual Functioning Questionnaire-Utility Index; WSE, Worse Seeing Eye | | | | | |

1. **Model stabilisation graphs**


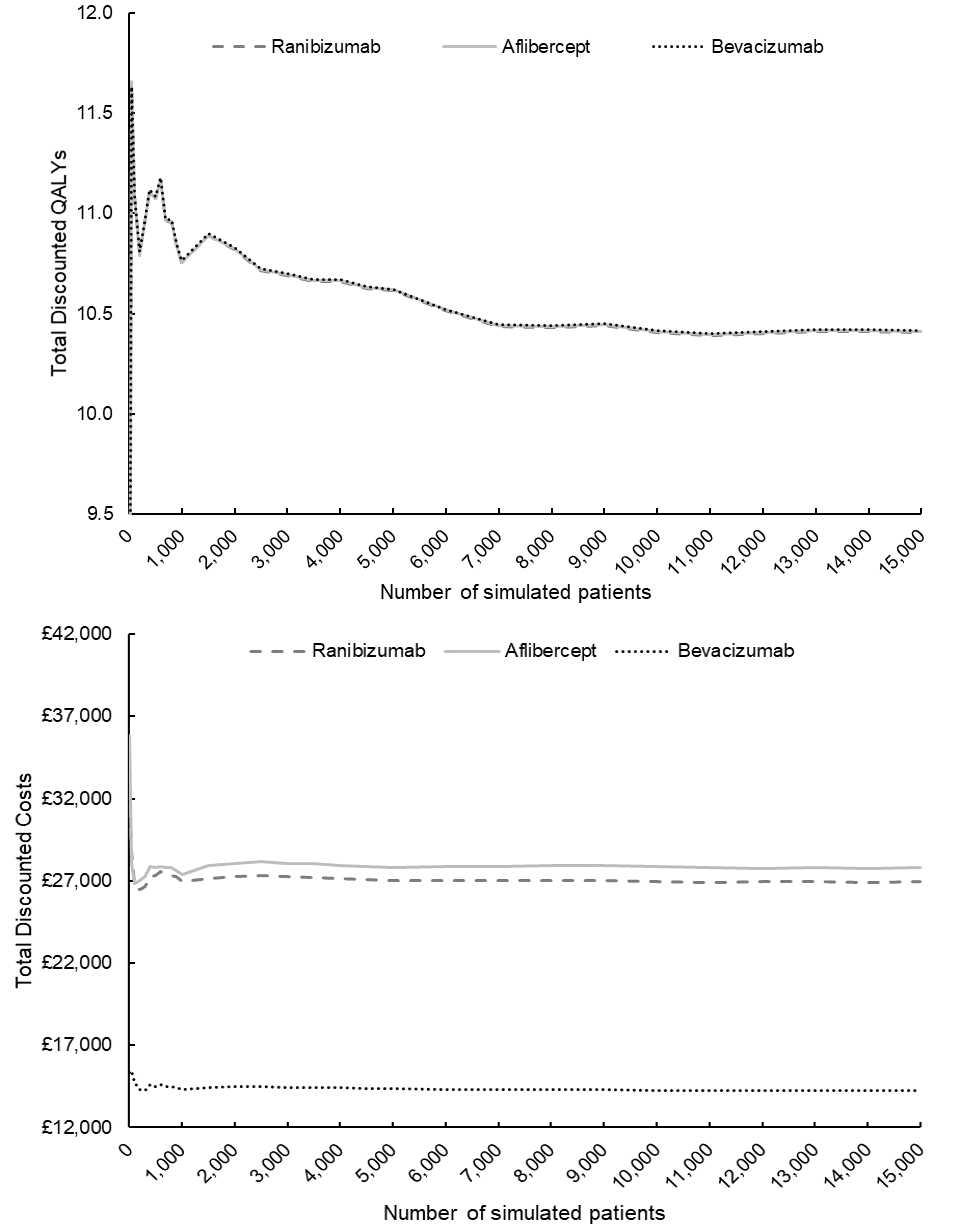


**References**

1. Latimer N. NICE DSU Technical Support Document 14: Undertaking survival analysis for economic evaluations alongside clinical trials - extrapolation with patient-level data. . 2011.

2. British National Formulary. Ranibizumab: solution for injection., 2019.

3. Royal Courts of Justice. Bayer v NHS Darlington CCG, Novartis v NHS Darlington CCG. . 2018.

4. NHS Improvement. Reference Costs. 2018.

5. NHS Improvement. Reference Costs. 2017.

6. Curtis LA BA. Unit Costs of Health and Social Care 2018., 2018.

7. Colquitt L JJ, Tan SC, Takeda A, Clegg AJ, Price A. Ranibizumab and pegaptanib for the treatment of age-related macular degeneration: a systematic review and economic evaluation. Health Technol Assess. 2008; 12.

8. Margrain TH. Minimising the impact of visual impairment. Low vision aids are a simple way of alleviating impairment. Bmj. 1999; 318: 1504.

9. Galaria, II, Casten RJ, Rovner BW. Development of a shorter version of the geriatric depression scale for visually impaired older patients. Int Psychogeriatr. 2000; 12: 435-43.

10. Longworth L, Yang Y, Young T, et al. Use of generic and condition-specific measures of health-related quality of life in NICE decision-making: a systematic review, statistical modelling and survey. Health Technol Assess. 2014; 18: 1-224.

11. Meads C, Hyde C. What is the cost of blindness? Br J Ophthalmol. 2003; 87: 1201-4.

12. National Institute for Health and Care Excellence. Adalimumab and dexamethasone for treating non-infectious uveitis [TA460]. 2017.

13. Bertelsen M, Linneberg A, Christoffersen N, et al. Mortality in patients with central retinal vein occlusion. Ophthalmology. 2014; 121: 637-42.

14. Klein BE KR, Moss SE. Change in visual acuity associated with cataract surgery. The Beaver Dam Eye Study. Ophthalmology. 1996; 103: 1727-31.
